# Strategies for Improving the Efficacy of CAR T Cells in Solid Cancers

**DOI:** 10.3390/cancers14030571

**Published:** 2022-01-23

**Authors:** Jon Amund Kyte

**Affiliations:** 1 Department of Cancer Immunology, Institute for Cancer Research, Oslo University Hospital, Mail Box 4950 Nydalen, 0424 Oslo, Norway; jonky@ous-hf.no; 2 Department of Clinical Cancer Research, Oslo University Hospital, Mail Box 4950 Nydalen, 0424 Oslo, Norway

**Keywords:** chimeric antigen receptor, cell therapy, solid cancers, cancer immunotherapy

## Abstract

**Simple Summary:**

Cell therapy with genetically retargeted T cells shows strong clinical efficacy against leukaemia and lymphoma. To make this therapy efficient against solid cancers, a series of hurdles must be addressed. This includes the need to enable the T cells to survive long term in patients and to overcome immunosuppressive mechanisms in the tumour. Further, it is essential to prevent tumour cells from escaping by losing the protein that is recognised by the infused cells. The present article provides an overview of the key strategies that are currently being investigated to overcome these hurdles. A series of approaches have been described in preclinical models, but these remain untested in patients. The further progress of the field will depend on evaluating more strategies in a proper clinical setting.

**Abstract:**

Therapy with T cells equipped with chimeric antigen receptors (CARs) shows strong efficacy against leukaemia and lymphoma, but not yet against solid cancers. This has been attributed to insufficient T cell persistence, tumour heterogeneity and an immunosuppressive tumour microenvironment. The present article provides an overview of key strategies that are currently investigated to overcome these hurdles. Basic aspects of CAR design are revisited, relevant for tuning the stimulatory signal to the requirements of solid tumours. Novel approaches for enhancing T cell persistence are highlighted, based on epigenetic or post-translational modifications. Further, the article describes CAR T strategies that are being developed for overcoming tumour heterogeneity and the escape of cancer stem cells, as well as for countering prevalent mechanisms of immune suppression in solid cancers. In general, personalised medicine is faced with a lack of drugs matching the patient’s profile. The advances and flexibility of modern gene engineering may allow for the filling of some of these gaps with tailored CAR T approaches addressing mechanisms identified as important in the individual patient. At this point, however, CAR T cell therapy remains unproved in solid cancers. The further progress of the field will depend on bringing novel strategies into clinical evaluation, while maintaining safety.

## 1. Introduction

T cells may be retargeted against cancer antigens by the use of chimeric antigen receptors (CARs). Therapy with CAR T cells shows strong clinical efficacy against haematological cancers [1], but not yet in solid tumours, except for individual cases [2]. The limited effect in solid cancers has been attributed to tumour heterogeneity, insufficient T cell persistence and an immunosuppressive tumour microenvironment (TME) [2,3]. Interestingly, therapy with tumour infiltrating lymphocytes can be highly effective against melanoma, suggesting a potential for cell therapy against solid cancers [4]. Additionally, some CAR studies have indicated tumour escape through antigen loss, even in solid cancers [5,6]. CAR T production involves isolating the patient’s T cells from blood, transducing with a gene encoding the CAR and giving them back to the patient [1]. In most CARs, the antigen binding part is a single-chain fragment variable (scFv) derived from a monoclonal antibody (mAb). The scFv is fused to a transmembrane domain and a signalling domain from the T cell receptor (TCR). The CAR concept is not dependent on a scFv-derived binding element, but only a few studies have employed other target-binding domains, such as natural ligands [7]. Compared to TCR, the CAR approach offers the advantage that T cell activation is independent of HLA type, allowing use across the patient population. The present review focuses on key challenges in the development of CAR T cell therapy against solid cancers, and on promising strategies to overcome these hurdles.

## 2. Co-Stimulatory Domains and Tuning of T Cell Stimulation

The breakthrough for CAR T cell therapy was only achieved after the introduction of second-generation CARs, that includes a co-stimulatory domain conferring the T cells with more potent effector functions [8]. This appears crucial for the persistence of CAR T cells in vivo, and for their clinical activity [2,9,10]. Third-generation CARs, with two co-stimulatory endodomains, have been available for more than a decade, but so far it is not known if this offers a clinical advantage. The most commonly used co-stimulatory CAR domains are derived from CD28 or 4-1BB, but other alternatives such as OX-40, GITR and ICOS have also been explored [11,12,13]. Most studies have been conducted with CD19 targeting CARs against haematological cancers. The optimal co-stimulatory domain may though differ between different cancer forms, target antigens and CAR constructs. A study in lymphoma and melanoma animal models compared the intracellular domains of CD28, Dap10, 4-1BB, GITR, ICOS, or OX40, using a chimeric receptor that targets tumor-associated PD1 ligands [13]. Here, they found that the Dap10 or 4-1BB co-stimulatory domains induced a preferential cytokine profile and T cell differentiation. There is also accumulating evidence from other studies, suggesting that 4-1BB protects T cells against exhaustion and activation-induced cell death, which is important for the long-term survival and activity of CAR T cells [9,10]. Philipson et al. used a CD19 CAR to study this question in a target-cell-free system, that may be informative also for solid cancers. They found improved ex vivo survival and subsequent expansion of 4-1BB-z CAR T cells when compared to CD28-z CAR T cells. Their data also pointed to a possible mechanism, as only 4-1BB CARs conferred a basic activation of non-canonical nuclear factor kappaB (ncNF-kappaB) signalling in the T cells. Further, the anti-CD19 4-1BB CAR enhanced ncNF-kappaB signalling after ligand engagement. In another important study, Sun et al. found that LCK, recruited into the synapse of a CD28-z CAR, caused antigen-independent CD3-z phosphorylation and increased T cell activation. By contrast, the synapse formed by a 4-1BB-z-CAR recruited the THEMIS-SHP1 phosphatase complex, that attenuates CD3-z phosphorylation. Sun et al. also investigated approaches to mitigate these issues, by engineering constructs to recruit LCK in 4-1BB CAR-T cells, or SHP1 in CD28 CAR T cells, and showed that these modifications had the desired functional effects. In another model, Zhao et al. demonstrated that CD28- and 4-1BB-mediated co-stimulation in CAR T cells confer different kinetics, as the CD28 endodomain promotes faster antitumor activity, compared to 4-1BB [11]. This observation is in line with a more glycolytic metabolism and higher susceptibility to exhaustion in CD28-mediated co-stimulation. By comparison, 4-1BB-mediated co-stimulation has been associated with a predominantly oxidative metabolism and lower susceptibility to exhaustion [14,15].

The 4-1BB-based second generation CARs have been successful in clinical use against haematological cancers. For solid tumours, issues with homing may delay the initial in vivo CAR-target recognition, and an immune-suppressive tumour environment may hinder their further expansion and persistence. Most likely, it is important to tune the activation signal, in order to balance potent antitumour activity with T cell persistence and memory. Sadelain and colleagues have explored if the activation potential of CD28-based CARs may be calibrated, in order to differentially reprogram T cell function and differentiation. In an elegant study, they found that CARs encoding a single immunoreceptor tyrosine-based activation motif directed T cells to different phenotypes [16]. The binding properties of the CAR scFv also influences the signalling levels, and may be tuned to facilitate prolonged T cell persistence [17]. These observations points to a possible strategy for durable memory combined with sufficient effector functionality. Again, the studies were performed with CD19 CARs. The signalling properties are dependent on the target and tumour form, and there is an evident need for similar studies to be conducted in solid cancers.

## 3. Strategies for Improved CAR T Cell Persistence

T cell exhaustion may limit the efficacy of CAR T therapy [2]. This challenge may to some extent be addressed by optimising the design of the CAR construct, as described above. Another key element is the protocol for CAR T transduction and ex vivo expansion. A prolonged time for cell expansion may be detrimental. This concern has limited the use of transposon-based transduction, which even in state-of-the-art facilities has given a lower transduction efficiency and depended on more sustained expansion of the transduced cells [18]. Improvements are though being made with the Sleeping Beauty transposon technology [19]. Most current clinical grade protocols for CAR T generation employ viral vectors and IL-2 as the only cytokine. However, multiple other cytokine combinations have been tested in experimental models, some of which have been reported to confer improved T cell survival and activity in vivo. This includes the use of IL-7, IL-15 and IL-21 during T cell expansion [20,21,22]. Though several of these alternatives have for many years been reported to be superior to the “IL-2 only” protocols, no optimal cytokine combination has been identified. The contradictory reports and relatively slow development of this fundamental aspect of CAR T research highlight the importance of publishing even unsuccessful attempts at improving protocols, to avoid a publication bias. The optimal cell generation protocol may depend on the microenvironment to which the cells should home. If optimal, standardised CAR T generation protocols for solid tumours can be determined, this will be valuable.

In 2021, two interesting reports emerged, demonstrating that epigenetic modification can prevent CAR T exhaustion, and even rejuvenate already “exhausted” T cells. Mackall’s group reported that the “resting” of CAR T cells with dasatinib, a clinically available tyrosine kinase inhibitor, prevented exhaustion of CAR T cells [23]. This worked both when applied during ex vivo CAR T expansion, and when administered as a drug in vivo. Likewise, Wang et al. reported that in vitro administration of decitabine, a DNA methyltransferase inhibitor, increased the functionality of CAR T cells both in vitro and in vivo [24]. Mackall’s group also investigated the use of a drug-regulatable on/off-switch, for transient inhibition of CAR surface expression. To this aim, a tonically signalling CAR was modified with a C-terminal destabilizing domain to enable drug-dependent control of CAR protein levels. The transient inhibition of CAR surface expression, and thereby pause in tonic CAR signalling, prevented cells from developing exhaustion, and redirected them to a memory-like phenotype. It is particularly interesting that these strategies for “resting” CAR T cells did not only work when applied before the cells became exhausted, but even restored functionality in phenotypically exhausted CAR T cells. This observation challenges the perception of T cell exhaustion as a fixed and final state, and suggests that it may be reversed. However, it remains unknown if this would apply also to T cells that have been “exhausted” for a longer period of time.

Post-translational modifications also offer opportunities for countering T cell exhaustion. In a pioneering study [25], Wei and colleagues studied regulatory RNase 1 (REGNASE-1) as a potential target for improving the persistence of effector CAR T cells. REGNASE-1 is an endoribonuclease that degrades RNA via internal cleavages and has been identified as an inhibitory mechanism hampering the effector properties of T cells [26,27], and to be involved in cancer [28]. Wei et al. found that the ablation of murine *REGNASE-1* in CD8+ CAR T cells prolonged their persistence in mouse models and improved the T cell accumulation in tumours. They also demonstrated that the *REGNASE-1*-deficient CAR T cells had enhanced anticancer efficacy in leukaemia and melanoma mouse models [25]. REGNASE-1 functions by targeting a series of specific mRNAs to degradation. Wei et al. found that the elimination of BATF (basic leucine zipper ATF-like transcription factor) suppressed the beneficial effects observed in REGNASE-1-deficient CD8+ CAR T cells [25]. Their study thus suggested that, in the context of CAR T therapy, a key target for REGNASE-1 is mRNA encoding BATF. This is in line with previous knowledge, as BATF is a transcription factor that is known to regulate the differentiation of several lymphocyte lineages, including CD4+ and CD8+ T cells [29,30], and a mediator of IL-12-induced Type-1 T cell differentiation. It has been reported that BATF is induced by IL-12, and via inhibition of Sirt1 leads to histone acetylation of the T-bet locus and increased production of ATP [31]. T-bet is a key transcription factor promoting Type-1 differentiation of both CD4+ and CD8+ T cells. It thus appears that the ablation of REGNASE-1 leads to the accumulation of BATF, thereby promoting the Type-1 differentiation of CAR T cells, leading to more potent antitumour activity. Further, the reported metabolic effects of BATF may explain why the persistence of CAR T cells was enhanced in the mouse models. This may be of particular importance to overcome other suppressive influences in a solid tumour microenvironment. However, it is worth noting that these effects of REGNASE-1 ablation on CAR T cells have to date only been reported in murine systems and remains to be confirmed in human T cells. Further, it should be underlined that REGNASE-1 silences a number of additional mRNAs, beyond BATF. This includes ICOS, OX40 and cytokines such as IL-2 and IL-6. Possibly, the ablation of REGNASE-1 may yield overly potent T cells and serious side effects. Still, the example of REGNASE illustrates the potential in looking into how the metabolic fitness of CAR T cells may be manipulated. The metabolic state of T cells has long been suggested to be important for the in vivo T cell survival, but the mechanisms have only been partially uncovered [32]. Possibly, increased knowledge on the specific mechanisms regulating T cell metabolism may lead to approaches that can be exploited for extending the persistence and function of CAR T cells in the solid tumour microenvironment.

## 4. Strategies for Countering Tumour Heterogeneity

Tumour heterogeneity, causing the escape of resistant cells, is a well-known challenge across cancer therapies and a particular hurdle for CAR T cells, which in principle only targets a single antigen. There is a lack of good CAR-targets in solid tumours, representing a fundamental roadblock. Of note, CD19 that is targeted in most currently approved CAR therapies, is not a tumour associated antigen, but a normal tissue differentiation antigen expressed both by malignant and normal B cells. The success of the CAR therapy against B cell malignancies is related to the fact that B cell deficiency is largely manageable, and that CD19 expression is relatively conserved among malignant B cells. In haematological cancers, the option of bone marrow transplant after CAR T therapy suggests that shared antigens expressed in both malignant and normal blood cells may be targeted. By contrast, the strategy of targeting a tissue-associated antigen, that is highly expressed in the corresponding normal tissue, is not applicable to most solid cancers. Prostate cancer represents a notable exception. Here, targets expressed even in normal prostate cells may be attractive, e.g., prostate stem cell antigen (PSCA), prostate-specific membrane antigen (PSMA) and six transmembrane epithelial antigen of the prostate 1 (STEAP1). Table 1 gives an overview over target antigens that are currently investigated in clinical trials in solid cancer forms.

Tumour escape may be countered by targeting proteins that are crucial to cancer development. Cancer initiating cells or so called cancer stem cells (CSCs) are a subpopulation of tumour cells that promote tumorigenesis, metastasis, relapse and escape from therapy [33]. Several reports have suggested that CSCs are not only capable of self-renewal, but also less sensitive to treatments [33,34,35]. The CSC-associated antigens are thus of huge importance to the cancer, and attractive targets for avoiding tumour escape. However, it is challenging to target CSCs without intolerable side effects, as CSC antigens are in general shared with normal stem cells. This applies to surface proteins such as CD44, CD133 and ALDH, as well as to intracellular CSC targets such as survivin and hTERT, which are being explored as targets for TCR-redirected cell therapy [36,37,38]. Another important point for target selection, is the issue of tumour evolution. This points to a need to target antigens that are frequently preserved after metastasis [39]. Unfortunately, the knowledge on antigen expression in metastatic lesions is generally limited, as most studies and databases rely on material from primary tumours. It is often ethically difficult to obtain biopsies from metastatic lesions, in particular larger tissue samples and fresh frozen biopsies, as this requires the use of invasive procedures, which are unlikely to provide any benefit for the individual patient. Systematic studies based on autopsies may address this issue, but are demanding to conduct appropriately in clinical practice. In spite of this data gap, it is well known that separate metastatic lesions in individual patients often vary in their antigen expression, and that different regions within each lesion may also show diversity [40,41].

**Figure 1:**
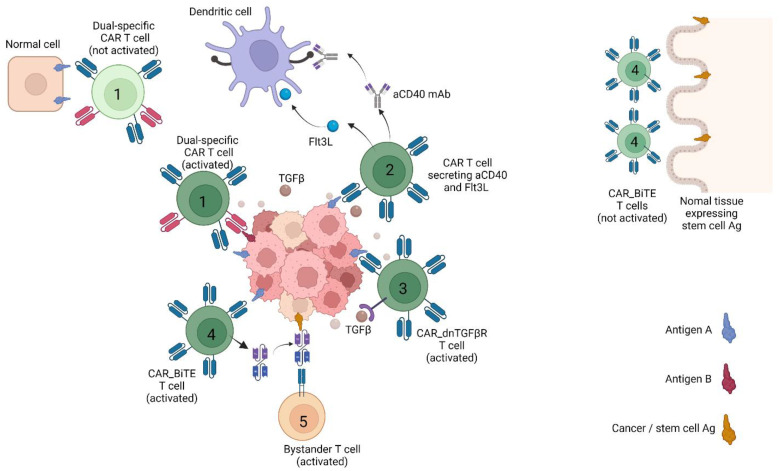
CAR T strategies countering heterogeneity and immune suppression in solid tumours. The figure depicts CAR T strategies that have been reported to work in animal models. T cells are labelled as follows: (**1**) Dual-receptor CAR T cells only activated in the presence of antigen A and B. (**2**) CAR T cell secreting the growth factor Flt3L or an agonist mAb against CD40, both of which stimulates dendritic cells. (**3**) CAR T cell resistant to TGFβ suppression due to double negative, non-signalling TGFβ receptor (dnTGFβ-R)**.** (**4**) T cell that upon CAR activation secretes a bispecific T cell engager (BiTE) against a cancer stem cell Ag also expressed by normal stem cell. (**5**) Bystander T cell with irrelevant specificity triggered by BiTE against cancer stem cell Ag. Created with BioRender.com. In addition, normal tissue expressing stem cell Ag was not discussed in main texts.

The combined targeting of several antigens represents an interesting approach for overcoming heterogeneity. Several groups have explored so-called “logical gating” strategies, where the T cells are triggered either by the combined expression of two antigens (A + B), or by the expression of antigen A, combined with a lack of antigen B (A − B). These approaches may allow for targeting antigens that are expressed both by tumour cells and normal cells, provided that the selected antigen combination (A + B or A − B) is tumour-specific (Figure 1). Kloss et al. pioneered the “A + B” approach, by using separate CARs for triggering CD3z and the co-stimulatory signal [42]. In a prostate cancer animal model, they demonstrated that tumour cells expressing both targets (PSMA and PSCA) were killed by the CAR T cells, while cells expressing only one of the targets were spared. Royal et al. developed another combinatorically activated system, in which a synthetic Notch receptor for antigen A induces the expression of a CAR for antigen B. The T cells were engineered to constitutively express a synNotch receptor that recognised antigen A. Furthermore, a separate gene encoding a CAR against antigen B was inserted into the T cell, but the expression of this gene was under the control of a promoter that required activation by the synNotch induced transcription factor. Upon recognition of Ag A, the synNotch receptor mediated the cleavage and release of the relevant transcription factor, and expression of the CAR against Ag B. Hence, the dual-receptor T cells were only armed and activated in the presence of tumour cells expressing both antigen A and B [43]. In an animal model with GFP and CD19 as target antigens, the authors demonstrated that the T cells efficiently eliminated combinatorial antigen tumours, while sparing cells/tumours expressing only a single antigen. To my knowledge, this very elegant principle has not yet been brought into clinical testing. It remains to be seen if the concept will work robustly in T cells produced with clinical manufacturing practice protocols, and how the functionality may be influenced by real-life expression levels of clinically relevant antigen combinations. Other groups are exploring a different strategy, whereby a CAR is used to trigger the local section of a tumour-targeting agent (Figure 1). Maus and colleagues have developed an interesting variant of this approach, for therapy of glioblastoma. They cloned a construct including a CAR specific for EGFRvIII, a glioblastoma-specific tumor antigen, that upon activation leads to the local secretion of a bispecific T cell engager (BiTE) against EGFR, an antigen frequently overexpressed in glioblastoma, but also expressed in normal tissues. This therapy showed efficacy in a preclinical model of glioblastoma [44].

Tumour heterogeneity may also be addressed by using the CAR T cells for triggering a host immune response against unknown antigens in the tumour. This strategy has been pursued by Lai et al., who engineered T cells to secrete Fms-like tyrosine kinase 3 ligand (Flt3L), which is a dendritic cell growth factor [45] (Figure 1). The Flt3L construct led to enhanced antitumour activity in solid tumour animal models, and to the induction of epitope spreading, i.e., reactivity towards antigens beyond those recognized by the CAR T cells. This approach is in line with old lessons learned from cancer vaccines, where epitope spreading has been linked to clinical efficacy [46]. A combination of CAR T cells with vaccines may also be pursued to cover a broader spectrum of targets [47].

## 5. Strategies for Improved Safety

Adoptive T cell therapy may produce severe side effects that are not always detectable in animal models or other preclinical test systems [48,49,50,51]. This applies even for on-target, off-tumour toxicity for antigens shown to be sufficiently safe for antibody-based therapies, such as HER2 [52]. Safety is a particular challenge when exploring novel CAR targets, as is needed for making advances against solid cancers. The above-mentioned A + B and A − B approaches offer the opportunity of targeting a wider range of antigens. However, the inherent unknowns of “first-in-man” clinical studies remain, as does the concern that some patients may develop side effects not observed in the majority. To address these safety concerns, several groups have developed remote control “suicide-mechanisms”, whereby the CAR T cells can be depleted if the patient develops severe side effects. Brenner and colleagues pioneered this strategy by developing a construct containing the CAR as well as iCasp9 [53]. In this system, the administration of AP1903 causes dimerization of iCasp9 within the CAR T cells and subsequent apoptosis, resulting in the specific depletion of the CAR T cells. An issue with suicide genes, is the expression difficulties of large constructs, which means that the addition of a suicide gene may limit the opportunity for other add-on features in the construct. Pule and colleagues have addressed this challenge, by developing a compact protein called RQR8, comprising a minimal binding epitope for rituximab, combined with a CD34-derived epitope that may be used as a marker and for clinical grade sorting of transduced cells. RQR8 is readily co-expressed with the CAR and allows for the depletion of CAR T cells with the mAb rituximab [54]. Such depletion may, however, not be complete. Most investigators employ vectors permanently integrating the receptor sequence into the T cell genome. Since these gene-modified T cells persist and expand in the patient, even a small population of remaining CAR T cells may expand and cause serious complications. Transient CAR expression based on mRNA transfection offers a safer route to clinical testing, and has been explored by us and others [12,36,55,56]. The advantage, and also the limitation, is that the mRNA-transfected T cells express the receptors only transiently. In our mRNA electroporation system, we demonstrated the eradication of leukaemia in a NOD SCID mouse model, amid minimal toxicity [12]. This approach overcomes safety concerns, eliminates expensive retrovirus production and simplifies the process for regulatory approval. The UPENN milieu has brought mRNA-based CARs into clinical testing, reporting possible clinical activity [55]. The efficacy of the mRNA approach is still unclear, as it has not been evaluated with CAR constructs known to be effective when used for viral, permanent retargeting.

## 6. Strategies for Overcoming Immune Suppression

A number of potent immunosuppressive mechanisms have been described in solid tumours and may counter the efficacy of CAR T cell therapy [3,57]. Tumour lesions frequently express inhibitory checkpoints such as PD-L1 and LAG-3, and are infiltrated by immunosuppressive cell populations, such as regulatory T cells, myeloid-derived suppressor cells, M2-macrophages and cancer-associated fibroblasts [3,57]. The cytokines TGFβ, IL-4 and IL-10 have been described as key mechanistic factors [3,57,58], as has adenosine-mediated suppression [59]. This knowledge points to a challenge, but also represents a rich opportunity for improving the efficacy of CAR T cell therapy. There are currently several ongoing clinical trials, mainly in heamatological malignancies, exploring the combination of CAR T cells with mAbs modulating PD-1/PD-L1 or other immune checkpoints. This is an attractive strategy, with a strong rational for synergy; the mAbs may enhance the effector functions of the CAR T cells, which may in turn trigger inflammation in the TME and make the tumour susceptible to checkpoint modulation. However, the clinical benefit of combing checkpoint modulating mAbs to CAR T cells remains unclear, as there are limited data from randomised trials.

A number of interesting strategies are investigated for harnessing the CAR T cells with add-on constructs, utilising technological advances in modern gene engineering [2]. This includes switch receptors combining PD-1 with a stimulatory intracellular domain [60,61], or engineered to secrete mAbs binding to agonist checkpoints such as CD40 [62] (Figure 1). Albelda and colleagues have addressed another key mechanism, adenosine-mediated immunosuppression. Adenosine and prostaglandin E2 (PGE2) activate protein kinase A (PKA), which inhibits TCR/CAR activation. This inhibition process depends on the localisation of PKA to the immune synapse, via binding to the membrane protein ezrin. Albelda and colleagues generated CAR T cells that expressed a small peptide that inhibits the association of PKA with ezrin, thus aborting the negative effects of PKA on CAR activation. The modified CAR T cells showed resistance to PGE2 and adenosine in vitro and increased antitumour activity in vivo [59].

Several groups have engineered CAR T cells to secrete pro-inflammatory cytokines such as IL-12, IL-15, IL-18 or IL-21, with promising results in animal models [63,64,65]. This may allow for using cytokines that are too toxic when administrated systemically, as their secretion can be triggered locally through CAR activation [66]. Still, systemic toxicity was observed in a pioneering clinical trial with tumour infiltrating lymphocytes that were engineered to secrete IL-12 [67], and the clinical benefit of CAR-associated cytokine secretion has not yet been established. A few clinical trials are currently ongoing in solid cancers, investigating the use of CAR T cells expressing IL-12 or IL-15 (Table 2).

TGFβ-mediated immunosuppression is important in most solid tumours [68,69,70]. Systemic targeting of TGFβ is difficult due to side effects, and several CAR T strategies for local countering of TGFβ-mediated suppression are being investigated [58]. Brenner’s group developed a double negative, non-signalling TGFβ receptor (dnTGFβ-R) [71] (Figure 1). The dnTGFβ-R approach is currently tested in clinical trials (Table 2), and a study has been completed in patients with castration-resistant metastatic prostate cancer (NCT03089203) [72]. Here, a PSA decline was observed in six out of ten patients, and PSA30 response occurred in four out of ten patients [73]. Chang et al. has pursued a different approach, developing a CAR that binds TGFβ by a scFv-binding domain and stimulates the engineered T cell through CD28 signaling [74]. The Th2 hallmark cytokine IL-4 may influence T cell differentiation and oppose CAR T activity [75]. To counter this, Vera and colleagues have developed a switch receptor converting IL-4 binding into stimulation, through the bridging of the IL-4 receptor exodomain to the IL-7 receptor endodomain. In a preclinical model of pancreatic cancer, they obtained promising data by combining this switch receptor with a hybrid receptor fusing the TGFβ receptor II exodomain with the endodomain of 4-1BB [75].

## 7. Perspectives on the Role of CAR T Cell Therapy and the Way Forward

Cancer is a moving target, escaping therapy through its adaptability. There is now considerable knowledge on how tumour heterogeneity, immune evasion and survival of so-called cancer stem cells mediate tumour escape. However, this knowledge has proved difficult to convert into improved therapy. As a result, metastatic solid cancers are still generally incurable. CAR T cell therapy has, in haematological malignancies, shown a remarkable capacity to cure disseminated and aggressive disease, and may hold a similar potential in metastatic solid cancers. A major hurdle for widespread application, is the fact that CAR T cell therapy is highly resource demanding. This highlights the need for simplified and automated production technologies, preferably de-centralised to the patient’s hospital. Still, this therapy is unlikely to be used for patients that can be cured by other treatments, and it is important to keep a firm focus on targets and mechanisms that are relevant in the metastatic and treatment refractory setting.

CAR T cell therapy has been pioneered in haematological cancers, and to a large extent with CARs against the CD19 target. This means that the most fundamental principles of CAR design rely on data from CD19 CARs, and have not been optimised for solid tumour targets. Ideally, there is a need for tailoring the co-stimulatory domain, spacer and binding affinity to the target epitope, and the expression levels on tumour cells. The same applies to other key factors. Possibly, many of the conclusions would remain, but important progress may be achieved through revisiting and challenging basic principles, in the appropriate solid tumour setting. The complexity and multiplicity of these interacting factors suggest that it is not feasible to test every combination in informative animal models, let alone in patients. To address this, artificial intelligence may be useful, in order to develop in silico algorithms and large-throughput in vitro screening assays, that may serve to select CAR constructs for advanced preclinical and clinical testing.

The field of CAR T therapy is currently fragmented, as many companies and research milieus conduct similar trials against the same targets, and in the same patient populations. The results from these studies are difficult to compare. A plethora of interesting strategies have been explored in preclinical studies, some of which have been described above. However, it remains largely unknown if these strategies would work in patients, and how they depend on the cancer form and other clinical variables. As mentioned, a few clinical trials have been initiated, exploring add-on features in CAR T cells, designed to improve the efficacy in solid cancers. A selection of interesting trials is listed in Table 2, all of which are phase 1 or phase I/II studies. The sparse number of clinical trials investigating novel features in CAR T cells is striking, compared to the plethora of attractive strategies that have shown efficacy in animal models. A key issue may be the fact that many approaches have been developed in model systems optimised to show the effect, often with CD19 CARs, while clinically relevant targets and the solid tumour microenvironment represent a very different challenge. Furthermore, CAR T production protocols optimised for research use may not easily be transferred to GMP. In order to bring the CAR T field forward, there is a need to address these hurdles, and for more clinical trials evaluating the novel concepts. In particular, randomised studies comparing specific alternatives in well-defined patient populations would be of substantial interest. Moreover, important insight may be gained from conducting personalised medicine studies in which strategies for countering patient-specific mechanisms of resistance are evaluated.

The CAR T approach is less restricted by host factors, compared to checkpoint inhibitors and vaccines, as the patient’s immune response is outsourced and generated in the laboratory. The approach allows us to design therapies utilising our knowledge on tumour biology and therapy escape, and even for personalising the treatment. In general, personalised medicine is faced with the lack of drugs matching the patient’s profile. The advances and flexibility of modern gene engineering may allow the filling of some of these gaps with tailored CAR T approaches addressing mechanisms identified as important in the individual patient. Possibly, the lack of perfect targets in solid tumours may be overcome by mapping the patient’s tumour antigen expression and microenvironment at baseline, and repeated mapping if therapy resistance develops. So far, there are few clinical studies pursuing this strategy, but with the increasing availability of CARs targeting different antigens and immunosuppressive mechanisms, studies comprising an a-la-carte menu of CARs may become doable.

## 8. Conclusions

CAR T cell therapy remains unproven in solid cancers. There is a need to overcome key hurdles, such as tumour heterogeneity, an immunosuppressive tumour microenvironment and insufficient T cell persistence. A series of approaches are being investigated to address these issues, with promising results in preclinical models. However, these concepts remain untested in patients and the leap from preclinical to clinical success should not be underestimated. The further progress of the field will depend on selecting the right strategies for clinical development and more effectively bringing these into proper clinical evaluation, while maintaining patient safety.

## Figures and Tables

**Table 1:** Target antigens investigated in ongoing or completed CAR T cell trials in solid cancers.

Antigen Target	Clinical Trial Identifiers
AXL	NCT03198052, NCT03393936, NCT05128786
B7-H3	NCT03198052, NCT04385173, NCT04185038, NCT04077866, NCT04483778, NCT04483778, NCT04432649, NCT04670068, NCT04077866
CAIX	NCT04969354
CD147	NCT03993743, NCT04045847
CD171	NCT02311621, NCT02311621, NCT02311621
CD20	NCT03893019
CD44v6	NCT04430595, NCT04427449
CD70	NCT02830724, NCT04438083
CEA	NCT03818165, NCT04348643, NCT03682744, NCT02850536, NCT04513431, NCT04037241, NCT01373047, NCT01212887, NCT02349724
CLDN18.2	NCT04404595, NCT04467853, NCT03874897, NCT04977193, NCT04966143, NCT03159819
CLDN6	NCT04503278
c-met	NCT01837602, NCT03060356
DLL3	NCT03392064
DR5	NCT03638206, NCT03941626
EGFRvIII	NCT03638206, NCT03941626, NCT02209376, NCT03726515, NCT03283631, NCT02664363, NCT01454596
EpCAM	NCT03563326, NCT03013712, NCT02915445, NCT04151186
ErbB	NCT01818323
FRα	NCT03585764, NCT03185468
GD2	NCT03356795, NCT04196413, NCT04539366, NCT02761915, NCT03373097, NCT02765243, NCT04099797, NCT03635632, NCT04430595, NCT03721068, NCT02992210, NCT01953900, NCT01822652, NCT05070156
gp100 (MHC-I)	NCT03649529
GFRα4	NCT04877613
GPC3	NCT03198052, NCT04506983, NCT03198546, NCT03198546, NCT04121273, NCT04377932, NCT02905188, NCT02932956, NCT03980288, NCT03884751, NCT05003895
HER2	NCT03198052, NCT03500991, NCT03696030, NCT04430595, NCT02442297, NCT04511871, NCT00902044, NCT01109095, NCT01935843
IL-13Rα2	NCT04510051, NCT02208362, NCT04661384
KLK2 kallikrein 2	NCT05022849
LeY	NCT03851146, NCT03198052
LFA1	NCT04420754
MMP2	NCT04214392
Mesothelin	NCT03198052, NCT03638206, NCT03356795, NCT03941626, NCT04503980, NCT04489862, NCT03747965, NCT03814447, NCT03916679, NCT03638193, NCT03799913, NCT03545815, NCT03497819, NCT03323944, NCT02414269, NCT03054298, NCT02792114, NCT01897415, NCT04981691, NCT03615313, NCT03054298, NCT02414269, NCT02792114
MUC1	NCT03198052, NCT03356795, NCT03633773, NCT03706326, NCT03525782, NCT04020575
MUC16	NCT03907527
MUC16ecto	NCT02498912
NECTIN4/FAP	NCT03932565
NKG2D	NCT03692429, NCT05131763
NKG2DL	NCT04270461, NCT04107142
PSCA	NCT03198052, NCT03873805, NCT02744287, NCT02744287
PSMA	NCT03356795, NCT04053062, NCT04227275, NCT03089203, NCT03185468, NCT04429451
ROR1	NCT02706392
ROR2	NCT03960060, NCT03393936
TM4SF1	NCT04151186
TnMUC1	NCT04025216

AXL, AXL receptor tyrosine kinase; CAR, chimeric antigen receptor; CEA, carcinoembryonic antigen; CLDN18.2, claudin 18 isoform 2; CLDN6, claudin 6; DLL3, delta-like canonical notch ligand 3; DR5, death receptor 5; EGFR, epidermal growth factor receptor; EGFRvIII, EGFR variant III; EpCAM, epithelial cell adhesion molecule; FAP, Fibroblast activation protein; FRα, folate receptor-α; GD2, disialoganglioside; gp100, glycoprotein 100; GPC3, glypican 3; HER2, human epidermal growth factor receptor 2; IL-13Rα2, interleukin 13 receptor α2; LeY, Lewis Y; LFA1, lymphocyte function-associated antigen 1; MHC-I, major histocompatibility complex class I; MMP2, matrix metalloproteinase 2; MUC1, mucin 1; MUC16ecto, mucin 16 ectodomain; NECTIN4, nectin cell adhesion molecule 4; NKG2D, natural killer group 2D; NKG2DL, natural killer group 2D ligand; PSCA, prostate stem cell antigen; PSMA, prostate-specific membrane antigen; ROR1, inactive tyrosine kinase transmembrane receptor 1; TM4SF1, transmembrane 4L six family member 1; TnMUC1, Tn glycoform of mucin 1.

**Table 2:** Selected CAR T cell trials in solid cancers exploring novel features.

Target Antigen	Clinical Trial Identifiers	Novel Feature
EGFR	NCT04153799	CAR T cells modified to express C-X-C Chemokine receptor type 5
	NCT03618381	CAR T cells directed at EGFR and CD19, based on the hypothesis that CD19+ B cells will promote CAR T persistence
	NCT03542799	CAR T with NFAT transcription factors inducing expression of IL—12
GPC3	NCT04377932	CAR T cells modified to secrete interleukin-15; safety/killing switch
MUC16	NCT03907527	CAR T cells expressing membrane-bound IL-15; safety/killing switch
MUC16ecto	NCT02498912	Intravenous and intraperitoneal infusion of CAR T cells modified to secrete IL-12
NECTIN4/FAP	NCT03932565	Intratumoural injection of Nectin4/FAP-targeted fourth-generation CAR T cells expressing IL7 and CCL19, or IL12
PSMA	NCT03089203	CAR T cells expressing dominant negative TGFβ receptor
	NCT04227275	CAR T cells expressing dominant negative TGFβ receptor

CAR, chimeric antigen receptor; EGFR, epidermal growth factor receptor; FAP, Fibroblast activation protein; GPC3, glypican 3; MUC1, mucin 16; MUC16ecto, mucin 16 ectodomain; NECTIN4, nectin cell adhesion molecule 4; PSMA, prostate-specific membrane antigen.
